# 2-(4-Meth­oxy­phen­oxy)-3-nitro­pyridine

**DOI:** 10.1107/S1600536810034057

**Published:** 2010-08-28

**Authors:** Shah Bakhtiar Nasir, Zanariah Abdullah, Zainal A. Fairuz, Seik Weng Ng, Edward R. T. Tiekink

**Affiliations:** a Department of Chemistry, University of Malaya, 50603 Kuala Lumpur, Malaysia

## Abstract

In the title mol­ecule, C_12_H_10_N_2_O_4_, the pyridine and benzene rings are almost orthogonal [dihedral angle = 86.69 (11)°], with the pyridine N atom directed towards the centre of the benzene ring. The –NO_2_ [O—N—C—C = −26.1 (3)°] and –OMe [C—O—C—C = 166.5 (2)°] substituents are not coplanar with their respective aromatic rings. In the crystal, supra­molecular layers in the *ab* plane are formed *via* C—H⋯π inter­actions involving methyl H atoms and the pyridine and benzene rings. Short N—O⋯π contacts (where the π-system is derived from the pyridine ring) occur between layers in the *c*-axis direction.

## Related literature

For background to fluorescence properties of compounds related to the title compound, see: Kawai *et al.* (2001 ▶); Abdullah (2005 ▶). For a related structure, see: Nasir *et al.* (2010 ▶).
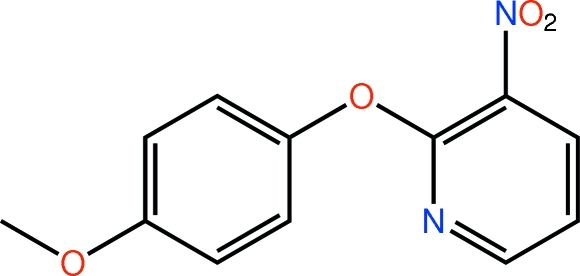


## Experimental

### 

#### Crystal data

C_12_H_10_N_2_O_4_
*M* *_r_* = 246.22
Orthorhombic, 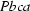

*a* = 7.4737 (10) Å
*b* = 12.8128 (17) Å
*c* = 24.529 (3) Å
*V* = 2348.8 (5) Å^3^
*Z* = 8
Mo *K*α radiation
μ = 0.11 mm^−1^
*T* = 293 K
0.30 × 0.28 × 0.07 mm

#### Data collection

Bruker SMART APEX CCD diffractometer
16986 measured reflections
2066 independent reflections
1364 reflections with *I* > 2σ(*I*)
*R*_int_ = 0.060

#### Refinement

*R*[*F* ^2^ > 2σ(*F* ^2^)] = 0.040
*wR*(*F* ^2^) = 0.123
*S* = 1.03
2066 reflections
165 parameters
H-atom parameters constrained
Δρ_max_ = 0.19 e Å^−3^
Δρ_min_ = −0.17 e Å^−3^

### 

Data collection: *APEX2* (Bruker, 2009 ▶); cell refinement: *SAINT* (Bruker, 2009 ▶); data reduction: *SAINT*; program(s) used to solve structure: *SHELXS97* (Sheldrick, 2008 ▶); program(s) used to refine structure: *SHELXL97* (Sheldrick, 2008 ▶); molecular graphics: *ORTEP-3* (Farrugia, 1997 ▶) and *DIAMOND* (Brandenburg, 2006 ▶); software used to prepare material for publication: *publCIF* (Westrip, 2010 ▶).

## Supplementary Material

Crystal structure: contains datablocks global, I. DOI: 10.1107/S1600536810034057/hb5614sup1.cif

Structure factors: contains datablocks I. DOI: 10.1107/S1600536810034057/hb5614Isup2.hkl

Additional supplementary materials:  crystallographic information; 3D view; checkCIF report

## Figures and Tables

**Table 1:** Hydrogen-bond geometry (Å, °) *Cg*1 and *Cg*2 are the centroids of the N1,C1–C5 and C6–C11 rings, respectively.

*D*—H⋯*A*	*D*—H	H⋯*A*	*D*⋯*A*	*D*—H⋯*A*
C12—H12a⋯*Cg*1^i^	0.96	2.73	3.521 (3)	140
C12—H12b⋯*Cg*2^ii^	0.96	2.80	3.616 (3)	143
N2—O1⋯*Cg*1^iii^	1.22 (1)	3.35 (1)	4.240 (2)	130 (1)

Symmetry codes: (i) 
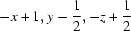
; (ii) 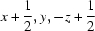; (iii) 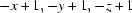.

**Fig. 1.**
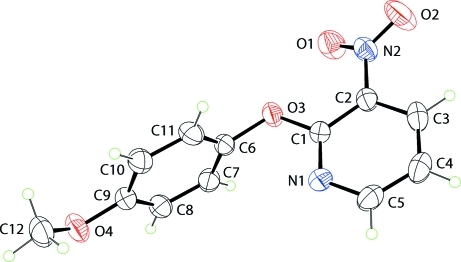
The molecular structure of (I) showing displacement ellipsoids at the 35% probability level.

**Fig. 2.**
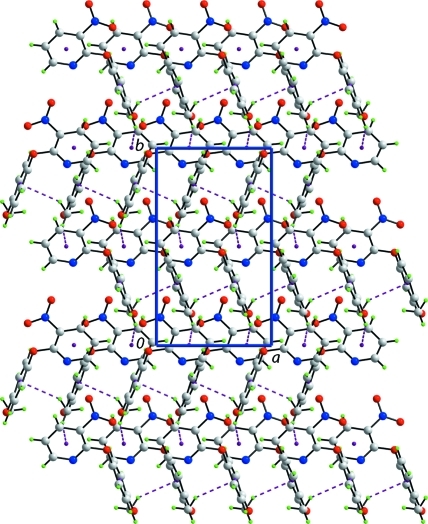
Supramolecular layer in the ab plane mediated by C–H···π interactions, shown as purple dashed lines, in the structure of (I).

**Fig. 3.**
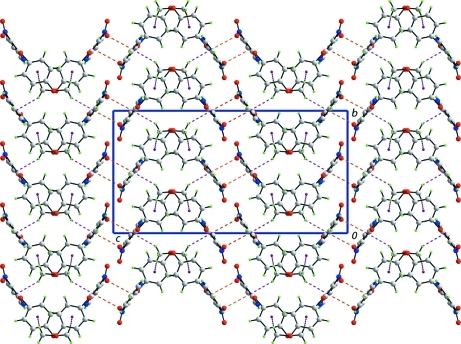
Unit-cell contents shown in projection down the a axis in (I), highlighting the stacking of layers along the c direction. The C–H···π and N–O···π interactions are shown as purple and orange dashed lines, respectively.

## supplementary crystallographic information

### Comment

Continued studies into the structural chemistry of *N*-heterocycle derivatives related to the title compound (Nasir *et al.*, 2010) arise owing to interest in examining their fluorescence properties (Kawai *et al.* 2001; Abdullah, 2005). It was in this context that the synthesis and characterization of the title compound, (I), was investigated.

In (I), Fig. 1, the pyridine ring is orthogonal to the benzene ring [dihedral angle = 86.69 (11) °] and is orientated so that the pyridine-N atom is directed towards the centre of the benzene ring. Whereas the methoxy group is deviates from co-planarity with the benzene ring to which it is connected [the C12–O4–C9–C8 torsion angle = 166.5 (2) °], the nitro group is even further twisted out of the plane of the pyridine ring [O1–N2–C2–C1 = -26.1 (3) °].

In the crystal packing, C–H..π and N–O···π interactions contribute to the stabilization of the structure. The C–H..π interactions involve methyl-H atoms interacting with the pyridine and benzene rings, and lead to the formation of layers in the *ab* plane, Fig. 2 and Table 1. The layers stack along the *c* axis and are connected by N–O···π contacts, where the π system is derived from the pyridine ring, Fig. 3 and Table 1.

### Experimental

4-Methoxyphenol (1.19 g, 96 mmol) was mixed with sodium hydroxide (0.384 g, 96 mmol) in several drops of water. The water was then evaporated. The paste was heated with 2-chloro-3-nitropyridine (1.49 g, 96 mmol) at 423–433 K for 5 h. The product was dissolved in water and the solution extracted with chloroform. The chloroform phase was dried over sodium sulfate; the evaporation of the solvent gave well shaped colourless blocks of (I).

### Refinement

Carbon-bound H-atoms were placed in calculated positions (C—H 0.93–0.96 Å) and were included in the refinement in the riding model approximation, with *U*_iso_(H) set to 1.2–1.5*U*_equiv_(C).

### Crystal data

**Table:**

C_12_H_10_N_2_O_4_	*F*(000) = 1024
*M**_r_* = 246.22	*D*_x_ = 1.393 Mg m^−^^3^
Orthorhombic, *P**b**c**a*	Mo *K*α radiation, λ = 0.71073 Å
Hall symbol: -P 2ac 2ab	Cell parameters from 2051 reflections
*a* = 7.4737 (10) Å	θ = 3.2–20.1°
*b* = 12.8128 (17) Å	µ = 0.11 mm^−^^1^
*c* = 24.529 (3) Å	*T* = 293 K
*V* = 2348.8 (5) Å^3^	Block, colourless
*Z* = 8	0.30 × 0.28 × 0.07 mm

### Data collection

**Table:**

Bruker SMART APEX CCD diffractometer	1364 reflections with *I* > 2σ(*I*)
Radiation source: fine-focus sealed tube	*R*_int_ = 0.060
graphite	θ_max_ = 25.0°, θ_min_ = 1.7°
ω scans	*h* = −8→8
16986 measured reflections	*k* = −15→15
2066 independent reflections	*l* = −27→29

### Refinement

**Table:**

Refinement on *F*^2^	Secondary atom site location: difference Fourier map
Least-squares matrix: full	Hydrogen site location: inferred from neighbouring sites
*R*[*F*^2^ > 2σ(*F*^2^)] = 0.040	H-atom parameters constrained
*wR*(*F*^2^) = 0.123	*w* = 1/[σ^2^(*F*_o_^2^) + (0.0589*P*)^2^ + 0.4422*P*] where *P* = (*F*_o_^2^ + 2*F*_c_^2^)/3
*S* = 1.03	(Δ/σ)_max_ = 0.001
2066 reflections	Δρ_max_ = 0.19 e Å^−^^3^
165 parameters	Δρ_min_ = −0.17 e Å^−^^3^
0 restraints	Extinction correction: *SHELXL97* (Sheldrick, 2008), Fc^*^=kFc[1+0.001xFc^2^λ^3^/sin(2θ)]^-1/4^
Primary atom site location: structure-invariant direct methods	Extinction coefficient: 0.0042 (9)

### Special details

**Table:**

Geometry. All e.s.d.'s (except the e.s.d. in the dihedral angle between two l.s. planes) are estimated using the full covariance matrix. The cell e.s.d.'s are taken into account individually in the estimation of e.s.d.'s in distances, angles and torsion angles; correlations between e.s.d.'s in cell parameters are only used when they are defined by crystal symmetry. An approximate (isotropic) treatment of cell e.s.d.'s is used for estimating e.s.d.'s involving l.s. planes.
Refinement. Refinement of *F*^2^ against ALL reflections. The weighted *R*-factor *wR* and goodness of fit *S* are based on *F*^2^, conventional *R*-factors *R* are based on *F*, with *F* set to zero for negative *F*^2^. The threshold expression of *F*^2^ > σ(*F*^2^) is used only for calculating *R*-factors(gt) *etc*. and is not relevant to the choice of reflections for refinement. *R*-factors based on *F*^2^ are statistically about twice as large as those based on *F*, and *R*- factors based on ALL data will be even larger.

### Fractional atomic coordinates and isotropic or equivalent isotropic displacement parameters (Å^2^)

**Table:**

	*x*	*y*	*z*	*U*_iso_*/*U*_eq_	
O1	0.6185 (3)	0.61280 (14)	0.47253 (8)	0.0772 (6)	
O2	0.4138 (3)	0.73025 (14)	0.47339 (9)	0.0986 (7)	
O3	0.56918 (18)	0.47091 (11)	0.39640 (7)	0.0603 (5)	
O4	0.8030 (2)	0.16173 (11)	0.25569 (7)	0.0659 (5)	
N1	0.2758 (2)	0.42013 (14)	0.38381 (8)	0.0536 (5)	
N2	0.4665 (3)	0.64262 (14)	0.46264 (8)	0.0553 (5)	
C1	0.3912 (3)	0.48503 (15)	0.40591 (9)	0.0440 (5)	
C2	0.3378 (3)	0.57022 (15)	0.43811 (8)	0.0450 (5)	
C3	0.1573 (3)	0.58629 (19)	0.44736 (9)	0.0578 (6)	
H3	0.1181	0.6424	0.4682	0.069*	
C4	0.0362 (3)	0.5166 (2)	0.42471 (11)	0.0654 (7)	
H4	−0.0861	0.5245	0.4303	0.078*	
C5	0.1010 (3)	0.4359 (2)	0.39382 (10)	0.0607 (6)	
H5	0.0192	0.3892	0.3789	0.073*	
C6	0.6196 (3)	0.39115 (16)	0.35921 (10)	0.0473 (6)	
C7	0.6735 (3)	0.29624 (17)	0.37948 (9)	0.0518 (6)	
H7	0.6684	0.2826	0.4167	0.062*	
C8	0.7358 (3)	0.22109 (16)	0.34344 (9)	0.0521 (6)	
H8	0.7739	0.1567	0.3566	0.063*	
C9	0.7416 (3)	0.24148 (15)	0.28798 (9)	0.0478 (6)	
C10	0.6896 (3)	0.33849 (16)	0.26848 (10)	0.0557 (6)	
H10	0.6960	0.3531	0.2314	0.067*	
C11	0.6281 (3)	0.41331 (16)	0.30457 (10)	0.0556 (6)	
H11	0.5926	0.4784	0.2918	0.067*	
C12	0.7750 (4)	0.1697 (2)	0.19822 (10)	0.0708 (8)	
H12A	0.8173	0.1074	0.1807	0.106*	
H12B	0.8393	0.2289	0.1843	0.106*	
H12C	0.6496	0.1783	0.1910	0.106*	

### Atomic displacement parameters (Å^2^)

**Table:**

	*U*^11^	*U*^22^	*U*^33^	*U*^12^	*U*^13^	*U*^23^
O1	0.0679 (12)	0.0787 (13)	0.0851 (14)	0.0010 (10)	−0.0152 (10)	−0.0238 (10)
O2	0.1079 (17)	0.0574 (11)	0.1304 (18)	0.0146 (11)	−0.0116 (14)	−0.0361 (11)
O3	0.0388 (9)	0.0600 (9)	0.0822 (12)	−0.0012 (7)	0.0057 (8)	−0.0320 (8)
O4	0.0852 (14)	0.0505 (9)	0.0620 (11)	0.0161 (8)	0.0111 (9)	−0.0123 (8)
N1	0.0424 (11)	0.0514 (11)	0.0669 (13)	−0.0061 (9)	0.0038 (9)	−0.0059 (9)
N2	0.0690 (14)	0.0490 (11)	0.0478 (12)	0.0041 (10)	0.0011 (10)	−0.0068 (9)
C1	0.0413 (12)	0.0424 (11)	0.0483 (13)	0.0008 (9)	0.0061 (10)	−0.0011 (9)
C2	0.0500 (13)	0.0437 (11)	0.0414 (12)	0.0054 (10)	0.0039 (10)	0.0027 (9)
C3	0.0606 (16)	0.0626 (14)	0.0503 (14)	0.0187 (13)	0.0118 (12)	0.0007 (11)
C4	0.0432 (14)	0.0796 (18)	0.0733 (18)	0.0092 (13)	0.0110 (13)	0.0079 (14)
C5	0.0426 (13)	0.0678 (15)	0.0717 (17)	−0.0073 (12)	0.0027 (12)	0.0039 (13)
C6	0.0353 (11)	0.0450 (12)	0.0616 (15)	−0.0023 (9)	0.0046 (10)	−0.0141 (10)
C7	0.0469 (13)	0.0571 (13)	0.0514 (14)	−0.0012 (10)	0.0048 (11)	−0.0043 (11)
C8	0.0534 (14)	0.0427 (11)	0.0604 (15)	0.0064 (10)	0.0017 (12)	0.0002 (11)
C9	0.0470 (13)	0.0431 (11)	0.0533 (14)	0.0015 (10)	0.0057 (11)	−0.0051 (10)
C10	0.0661 (16)	0.0468 (12)	0.0540 (15)	0.0027 (11)	0.0072 (12)	0.0020 (10)
C11	0.0587 (15)	0.0386 (11)	0.0694 (17)	0.0041 (10)	0.0021 (13)	−0.0019 (11)
C12	0.0761 (19)	0.0777 (17)	0.0585 (17)	0.0018 (14)	0.0073 (14)	−0.0230 (13)

### Geometric parameters (Å, °)

**Table:**

O1—N2	1.223 (2)	C5—H5	0.9300
O2—N2	1.219 (2)	C6—C11	1.371 (3)
O3—C1	1.363 (3)	C6—C7	1.374 (3)
O3—C6	1.421 (2)	C7—C8	1.388 (3)
O4—C9	1.372 (2)	C7—H7	0.9300
O4—C12	1.429 (3)	C8—C9	1.386 (3)
N1—C1	1.315 (3)	C8—H8	0.9300
N1—C5	1.345 (3)	C9—C10	1.387 (3)
N2—C2	1.466 (3)	C10—C11	1.384 (3)
C1—C2	1.405 (3)	C10—H10	0.9300
C2—C3	1.383 (3)	C11—H11	0.9300
C3—C4	1.387 (3)	C12—H12A	0.9600
C3—H3	0.9300	C12—H12B	0.9600
C4—C5	1.371 (3)	C12—H12C	0.9600
C4—H4	0.9300		
			
C1—O3—C6	117.66 (15)	C7—C6—O3	118.8 (2)
C9—O4—C12	117.84 (18)	C6—C7—C8	118.8 (2)
C1—N1—C5	117.85 (19)	C6—C7—H7	120.6
O2—N2—O1	123.0 (2)	C8—C7—H7	120.6
O2—N2—C2	117.4 (2)	C7—C8—C9	120.3 (2)
O1—N2—C2	119.57 (18)	C7—C8—H8	119.8
N1—C1—O3	119.05 (18)	C9—C8—H8	119.8
N1—C1—C2	122.5 (2)	O4—C9—C10	124.2 (2)
O3—C1—C2	118.47 (18)	O4—C9—C8	115.89 (19)
C3—C2—C1	119.0 (2)	C10—C9—C8	119.90 (19)
C3—C2—N2	118.59 (19)	C11—C10—C9	119.6 (2)
C1—C2—N2	122.40 (19)	C11—C10—H10	120.2
C2—C3—C4	118.3 (2)	C9—C10—H10	120.2
C2—C3—H3	120.8	C6—C11—C10	119.8 (2)
C4—C3—H3	120.8	C6—C11—H11	120.1
C5—C4—C3	118.5 (2)	C10—C11—H11	120.1
C5—C4—H4	120.8	O4—C12—H12A	109.5
C3—C4—H4	120.8	O4—C12—H12B	109.5
N1—C5—C4	123.9 (2)	H12A—C12—H12B	109.5
N1—C5—H5	118.1	O4—C12—H12C	109.5
C4—C5—H5	118.1	H12A—C12—H12C	109.5
C11—C6—C7	121.54 (19)	H12B—C12—H12C	109.5
C11—C6—O3	119.39 (19)		
			
C5—N1—C1—O3	−180.0 (2)	C3—C4—C5—N1	−0.3 (4)
C5—N1—C1—C2	−1.4 (3)	C1—O3—C6—C11	86.8 (2)
C6—O3—C1—N1	5.0 (3)	C1—O3—C6—C7	−98.8 (2)
C6—O3—C1—C2	−173.55 (19)	C11—C6—C7—C8	−0.8 (3)
N1—C1—C2—C3	0.5 (3)	O3—C6—C7—C8	−175.10 (18)
O3—C1—C2—C3	179.02 (19)	C6—C7—C8—C9	−0.6 (3)
N1—C1—C2—N2	−179.89 (19)	C12—O4—C9—C10	−14.5 (3)
O3—C1—C2—N2	−1.4 (3)	C12—O4—C9—C8	166.5 (2)
O2—N2—C2—C3	−24.8 (3)	C7—C8—C9—O4	−179.2 (2)
O1—N2—C2—C3	153.5 (2)	C7—C8—C9—C10	1.8 (3)
O2—N2—C2—C1	155.6 (2)	O4—C9—C10—C11	179.4 (2)
O1—N2—C2—C1	−26.1 (3)	C8—C9—C10—C11	−1.6 (3)
C1—C2—C3—C4	0.6 (3)	C7—C6—C11—C10	1.0 (3)
N2—C2—C3—C4	−179.1 (2)	O3—C6—C11—C10	175.26 (19)
C2—C3—C4—C5	−0.6 (4)	C9—C10—C11—C6	0.2 (4)
C1—N1—C5—C4	1.4 (4)		

### Hydrogen-bond geometry (Å, °)

**Table:**

Cg1 and Cg2 are the centroids of the N1,C1–C5 and C6–C11 rings, respectively.

**Table:**

*D*—H···*A*	*D*—H	H···*A*	*D*···*A*	*D*—H···*A*
C12—H12a···Cg1^i^	0.96	2.73	3.521 (3)	140
C12—H12b···Cg2^ii^	0.96	2.80	3.616 (3)	143
N2—O1···Cg1^iii^	1.223 (2)	3.350 (2)	4.240 (2)	129.93 (15)

Symmetry codes: (i) −*x*+1, *y*−1/2, −*z*+1/2; (ii) *x*+1/2, *y*, −*z*+1/2; (iii) −*x*+1, −*y*+1, −*z*+1.
